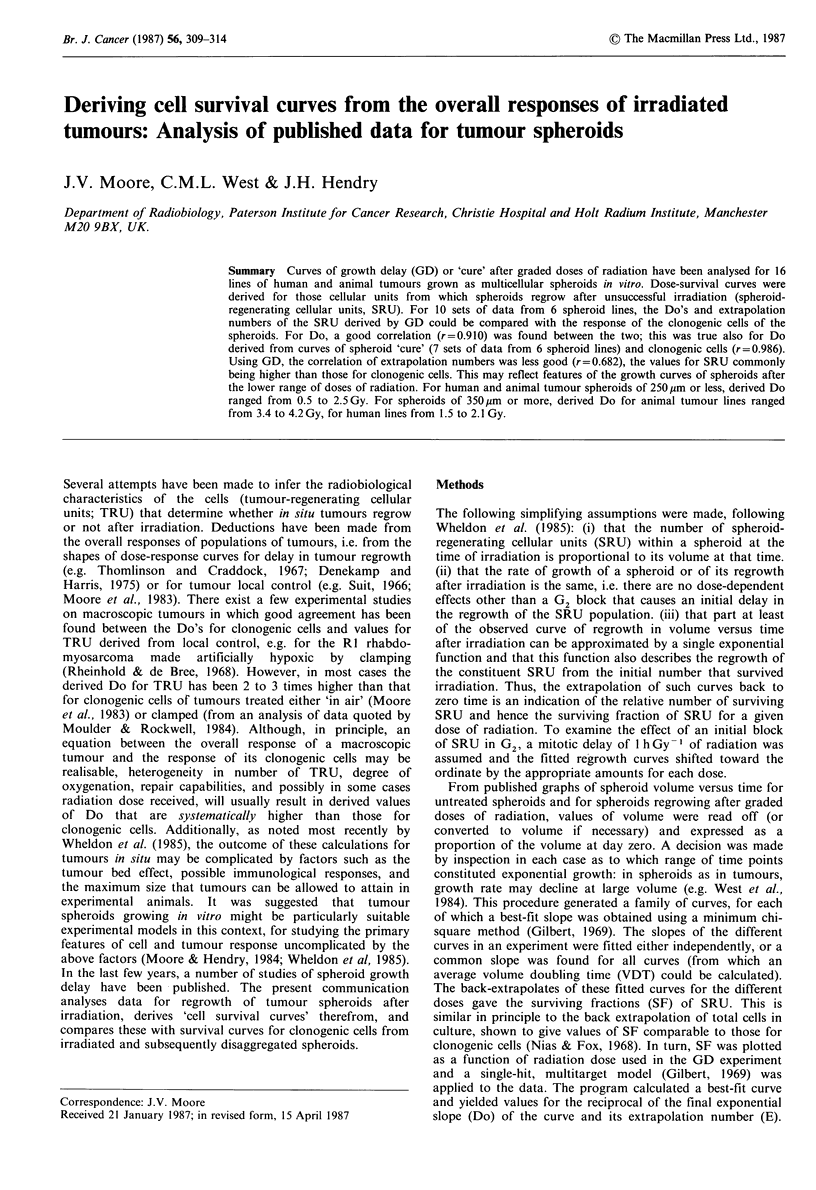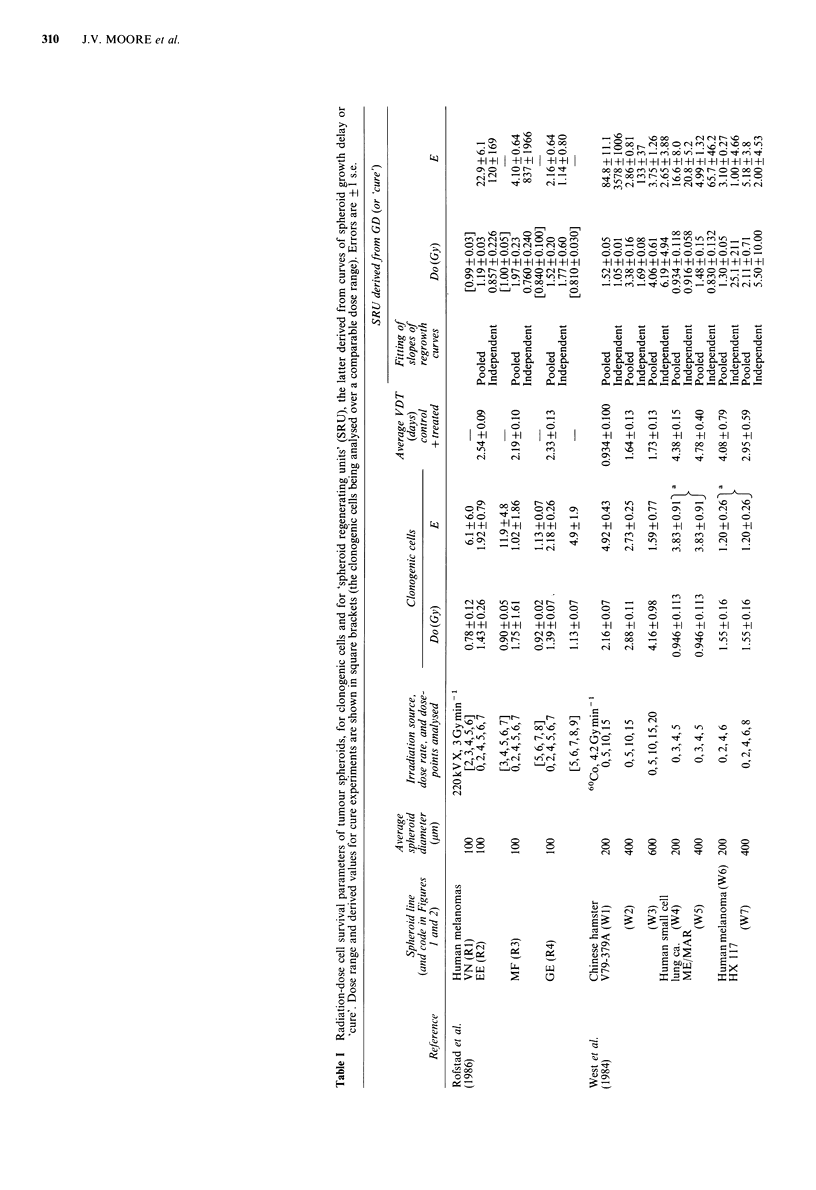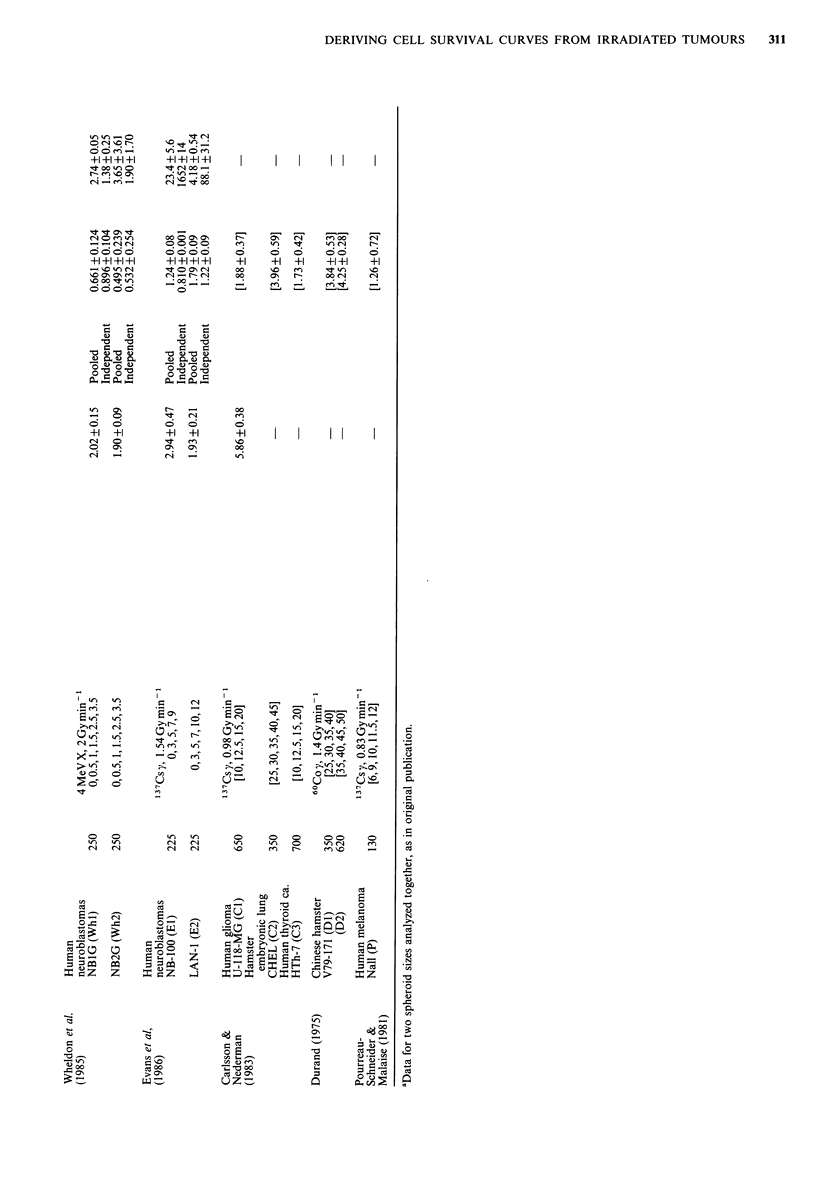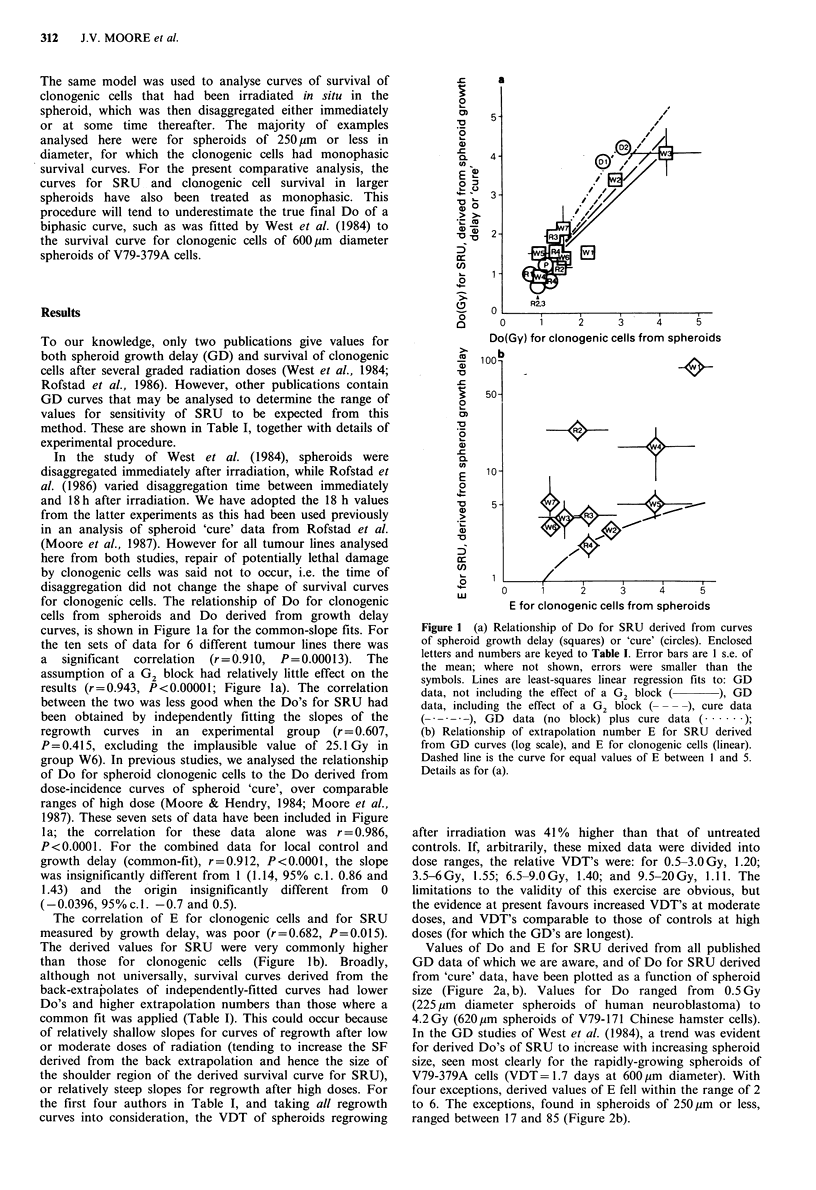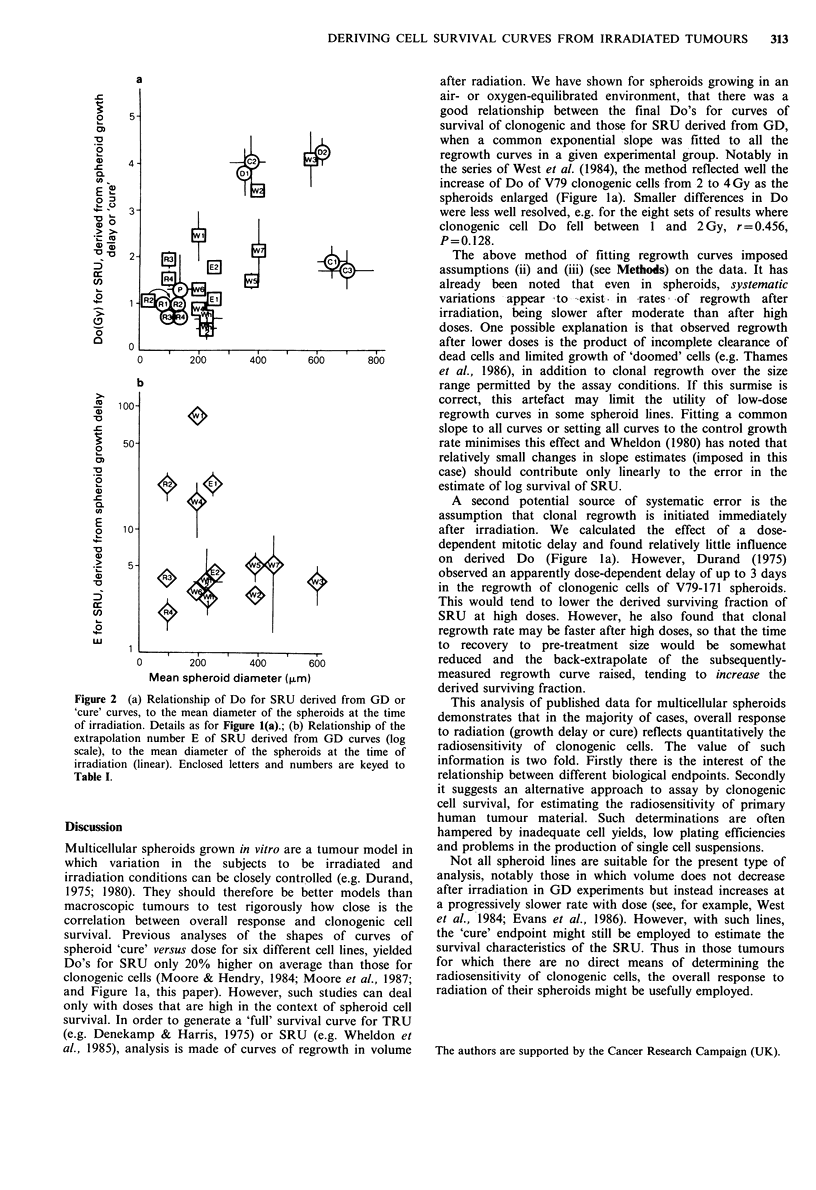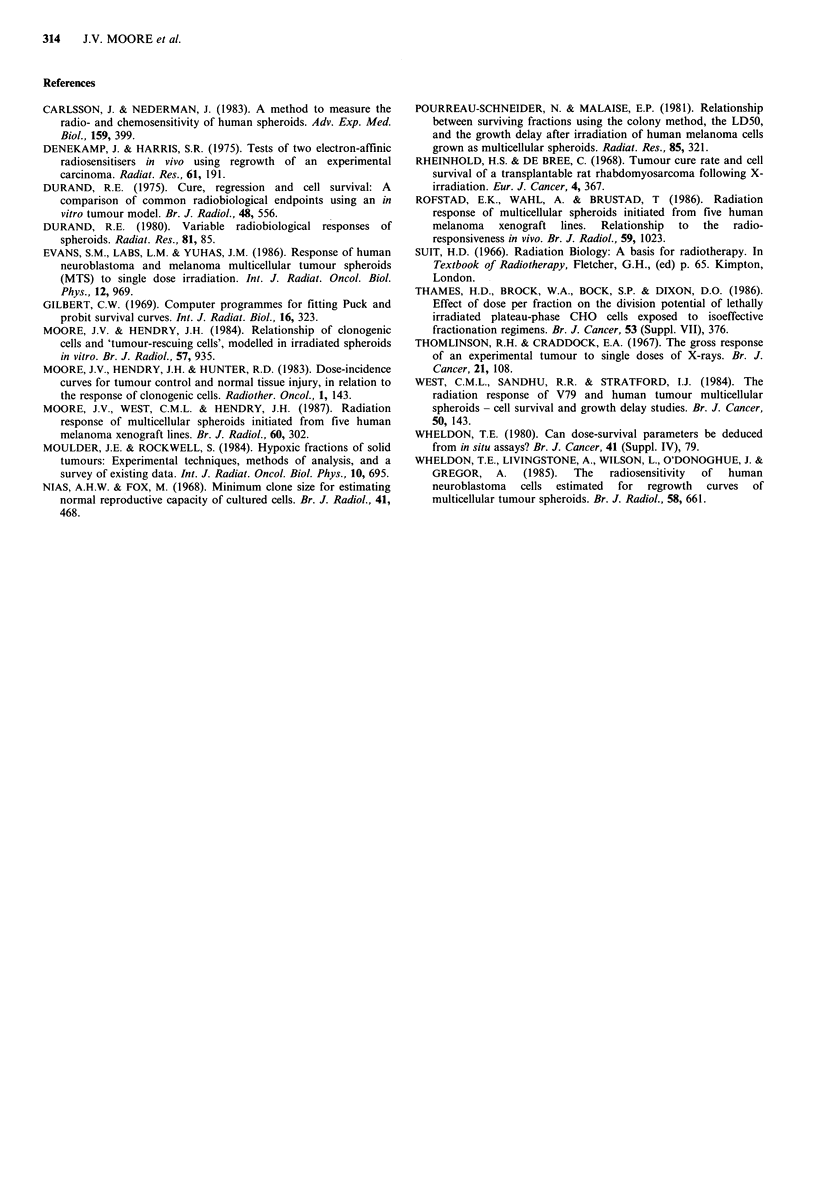# Deriving cell survival curves from the overall responses of irradiated tumours: analysis of published data for tumour spheroids.

**DOI:** 10.1038/bjc.1987.194

**Published:** 1987-09

**Authors:** J. V. Moore, C. M. West, J. H. Hendry

**Affiliations:** Department of Radiobiology, Paterson Institute for Cancer Research, Christie Hospital and Holt Radium Institute, Manchester, UK.

## Abstract

Curves of growth delay (GD) or 'cure' after graded doses of radiation have been analysed for 16 lines of human and animal tumours grown as multicellular spheroids in vitro. Dose-survival curves were derived for those cellular units from which spheroids regrow after unsuccessful irradiation (spheroid-regenerating cellular units, SRU). For 10 sets of data from 6 spheroid lines, the Do's and extrapolation numbers of the SRU derived by GD could be compared with the response of the clonogenic cells of the spheroids. For Do, a good correlation (r = 0.910) was found between the two; this was true also for Do derived from curves of spheroid 'cure' (7 sets of data from 6 spheroid lines) and clonogenic cells (r = 0.986). Using GD, the correlation of extrapolation numbers was less good (r = 0.682), the values for SRU commonly being higher than those for clonogenic cells. This may reflect features of the growth curves of spheroids after the lower range of doses of radiation. For human and animal tumour spheroids of 250 microns or less, derived Do ranged from 0.5 to 2.5 Gy. For spheroids of 350 microns or more, derived Do for animal tumour lines ranged from 3.4 to 4.2 Gy, for human lines from 1.5 to 2.1 Gy.


*Br. J. Cancer* (1987) **56**, 309–314 © The Macmillan Press Ltd., 1987

### Deriving cell survival curves from the overall responses of irradiated tumours: Analysis of published data for tumour spheroids

J.V. Moore, C.M.L. West & J.H. Hendry

*Department of Radiobiology, Paterson Institute for Cancer Research, Christie Hospital and Holt Radium Institute, Manchester M20 9BX, UK.*

**Summary** Curves of growth delay (GD) or 'cure' after graded doses of radiation have been analysed for 16 lines of human and animal tumours grown as multicellular spheroids *in vitro*. Dose-survival curves were derived for those cellular units from which spheroids regrow after unsuccessful irradiation (spheroid-regenerating cellular units, SRU). For 10 sets of data from 6 spheroid lines, the Do's and extrapolation numbers of the SRU derived by GD could be compared with the response of the clonogenic cells of the spheroids. For Do, a good correlation ($r = 0.910$) was found between the two; this was true also for Do derived from curves of spheroid 'cure' (7 sets of data from 6 spheroid lines) and clonogenic cells ($r = 0.986$). Using GD, the correlation of extrapolation numbers was less good ($r = 0.682$), the values for SRU commonly being higher than those for clonogenic cells. This may reflect features of the growth curves of spheroids after the lower range of doses of radiation. For human and animal tumour spheroids of 250 $\mu$m or less, derived Do ranged from 0.5 to 2.5 Gy. For spheroids of 350 $\mu$m or more, derived Do for animal tumour lines ranged from 3.4 to 4.2 Gy, for human lines from 1.5 to 2.1 Gy.

Several attempts have been made to infer the radiobiological characteristics of the cells (tumour-regenerating cellular units; TRU) that determine whether *in situ* tumours regrow or not after irradiation. Deductions have been made from the overall responses of populations of tumours, i.e. from the shapes of dose-response curves for delay in tumour regrowth (e.g. Thomlinson and Craddock, 1967; Denekamp and Harris, 1975) or for tumour local control (e.g. Suit, 1966; Moore *et al.*, 1983). There exist a few experimental studies on macroscopic tumours in which good agreement has been found between the Do's for clonogenic cells and values for TRU derived from local control, e.g. for the R1 rhabdomyosarcoma made artificially hypoxic by clamping (Rheinhold & de Bree, 1968). However, in most cases the derived Do for TRU has been 2 to 3 times higher than that for clonogenic cells of tumours treated either 'in air' (Moore *et al.*, 1983) or clamped (from an analysis of data quoted by Moulder & Rockwell, 1984). Although, in principle, an equation between the overall response of a macroscopic tumour and the response of its clonogenic cells may be realisable, heterogeneity in number of TRU, degree of oxygenation, repair capabilities, and possibly in some cases radiation dose received, will usually result in derived values of Do that are *systematically* higher than those for clonogenic cells. Additionally, as noted most recently by Wheldon *et al.* (1985), the outcome of these calculations for tumours *in situ* may be complicated by factors such as the tumour bed effect, possible immunological responses, and the maximum size that tumours can be allowed to attain in experimental animals. It was suggested that tumour spheroids growing *in vitro* might be particularly suitable experimental models in this context, for studying the primary features of cell and tumour response uncomplicated by the above factors (Moore & Hendry, 1984; Wheldon *et al*, 1985). In the last few years, a number of studies of spheroid growth delay have been published. The present communication analyses data for regrowth of tumour spheroids after irradiation, derives 'cell survival curves' therefrom, and compares these with survival curves for clonogenic cells from irradiated and subsequently disaggregated spheroids.

Correspondence: J.V. Moore
Received 21 January 1987; in revised form, 15 April 1987

### Methods

The following simplifying assumptions were made, following Wheldon *et al.* (1985): (i) that the number of spheroid-regenerating cellular units (SRU) within a spheroid at the time of irradiation is proportional to its volume at that time. (ii) that the rate of growth of a spheroid or of its regrowth after irradiation is the same, i.e. there are no dose-dependent effects other than a $G_2$ block that causes an initial delay in the regrowth of the SRU population. (iii) that part at least of the observed curve of regrowth in volume versus time after irradiation can be approximated by a single exponential function and that this function also describes the regrowth of the constituent SRU from the initial number that survived irradiation. Thus, the extrapolation of such curves back to zero time is an indication of the relative number of surviving SRU and hence the surviving fraction of SRU for a given dose of radiation. To examine the effect of an initial block of SRU in $G_2$, a mitotic delay of 1 h $Gy^{-1}$ of radiation was assumed and the fitted regrowth curves shifted toward the ordinate by the appropriate amounts for each dose.

From published graphs of spheroid volume versus time for untreated spheroids and for spheroids regrowing after graded doses of radiation, values of volume were read off (or converted to volume if necessary) and expressed as a proportion of the volume at day zero. A decision was made by inspection in each case as to which range of time points constituted exponential growth: in spheroids as in tumours, growth rate may decline at large volume (e.g. West *et al.*, 1984). This procedure generated a family of curves, for each of which a best-fit slope was obtained using a minimum chi-square method (Gilbert, 1969). The slopes of the different curves in an experiment were fitted either independently, or a common slope was found for all curves (from which an average volume doubling time (VDT) could be calculated). The back-extrapolates of these fitted curves for the different doses gave the surviving fractions (SF) of SRU. This is similar in principle to the back extrapolation of total cells in culture, shown to give values of SF comparable to those for clonogenic cells (Nias & Fox, 1968). In turn, SF was plotted as a function of radiation dose used in the GD experiment and a single-hit, multitarget model (Gilbert, 1969) was applied to the data. The program calculated a best-fit curve and yielded values for the reciprocal of the final exponential slope (Do) of the curve and its extrapolation number (E).

**Table I** Radiation-dose cell survival parameters of tumour spheroids, for clonogenic cells and for 'spheroid regenerating units' (SRU), the latter derived from curves of spheroid growth delay or 'cure'. Dose range and derived values for cure experiments are shown in square brackets (the clonogenic cells being analysed over a comparable dose range). Errors are ±1 s.e.

| *Reference* | *Spheroid line (and code in Figures 1 and 2)* | *Average spheroid diameter (μm)* | *Irradiation source, dose rate, and dose-points analysed* | *Clonogenic cells* | | *Average VDT (days) control + treated* | *SRU derived from GD (or 'cure')* | | |
|---|---|---|---|---|---|---|---|---|---|
| | | | | *Do (Gy)* | *E* | | *Fitting of slopes of regrowth curves* | *Do (Gy)* | *E* |
| Rofstad *et al.* | Human melanomas | | 220 kV X, 3 Gy min$^{-1}$ | | | | | | |
| (1986) | VN (R1) | 100 | [2, 3, 4, 5, 6] | 0.78±0.12 | 6.1±6.0 | — | | [0.99±0.03] | |
| | EE (R2) | 100 | 0, 2, 4, 5, 6, 7 | 1.43±0.26 | 1.92±0.79 | 2.54±0.09 | Pooled | 1.19±0.03 | 22.9±6.1 |
| | | | | | | | Independent | 0.857±0.226 | 120±169 |
| | | | [3, 4, 5, 6, 7] | 0.90±0.05 | 11.9±4.8 | — | | [1.00±0.05] | — |
| | MF (R3) | 100 | 0, 2, 4, 5, 6, 7 | 1.75±1.61 | 1.02±1.86 | 2.19±0.10 | Pooled | 1.97±0.23 | 4.10±0.64 |
| | | | | | | | Independent | 0.760±0.240 | 837±1966 |
| | | | [5, 6, 7, 8] | 0.92±0.02 | 1.13±0.07 | — | | [0.840±0.100] | — |
| | GE (R4) | 100 | 0, 2, 4, 5, 6, 7 | 1.39±0.07 | 2.18±0.26 | 2.33±0.13 | Pooled | 1.52±0.20 | 2.16±0.64 |
| | | | | | | | Independent | 1.77±0.60 | 1.14±0.80 |
| | | | [5, 6, 7, 8, 9] | 1.13±0.07 | 4.9±1.9 | — | | [0.810±0.030] | — |
| West *et al.* | Chinese hamster | | $^{60}$Co, 4.2 Gy min$^{-1}$ | | | | | | |
| (1984) | V79-379A (W1) | 200 | 0, 5, 10, 15 | 2.16±0.07 | 4.92±0.43 | 0.934±0.100 | Pooled | 1.52±0.05 | 84.8±11.1 |
| | | | | | | | Independent | 1.05±0.01 | 3578±1006 |
| | (W2) | 400 | 0, 5, 10, 15 | 2.88±0.11 | 2.73±0.25 | 1.64±0.13 | Pooled | 3.38±0.16 | 2.86±0.81 |
| | | | | | | | Independent | 1.69±0.08 | 133±37 |
| | (W3) | 600 | 0, 5, 10, 15, 20 | 4.16±0.98 | 1.59±0.77 | 1.73±0.13 | Pooled | 4.06±0.61 | 3.75±1.26 |
| | Human small cell | | | | | | Independent | 6.19±4.94 | 2.65±3.88 |
| | lung ca. (W4) | 200 | 0, 3, 4, 5 | 0.946±0.113 | 3.83±0.91 [a] | 4.38±0.15 | Pooled | 0.934±0.118 | 16.6±8.0 |
| | ME/MAR | | | | | | Independent | 0.916±0.058 | 20.8±5.2 |
| | (W5) | 400 | 0, 3, 4, 5 | 0.946±0.113 | 3.83±0.91 | 4.78±0.40 | Pooled | 1.48±0.15 | 4.99±1.32 |
| | | | | | | | Independent | 0.830±0.132 | 65.7±46.2 |
| | Human melanoma (W6) | 200 | 0, 2, 4, 6 | 1.55±0.16 | 1.20±0.26 [a] | 4.08±0.79 | Pooled | 1.30±0.05 | 3.10±0.27 |
| | HX 117 | | | | | | Independent | 25.1±211 | 1.00±4.66 |
| | (W7) | 400 | 0, 2, 4, 6, 8 | 1.55±0.16 | 1.20±0.26 | 2.95±0.59 | Pooled | 2.11±0.71 | 5.18±3.8 |
| | | | | | | | Independent | 5.50±10.00 | 2.00±4.53 |

| | | | | | | | |
|---|---|---|---|---|---|---|---|
| Wheldon *et al.* (1985) | Human neuroblastomas | | 4 MeV X, 2 Gy min$^{-1}$ | | | | |
| | NB1G (Wh1) | 250 | 0, 0.5, 1, 1.5, 2.5, 3.5 | 2.02±0.15 | Pooled | 0.661±0.124 | 2.74±0.05 |
| | | | | | Independent | 0.896±0.104 | 1.38±0.25 |
| | NB2G (Wh2) | 250 | 0, 0.5, 1, 1.5, 2.5, 3.5 | 1.90±0.09 | Pooled | 0.495±0.239 | 3.65±3.61 |
| | | | | | Independent | 0.532±0.254 | 1.90±1.70 |
| Evans *et al*, (1986) | Human neuroblastomas | | $^{137}Cs\ \gamma$, 1.54 Gy min$^{-1}$ | | | | |
| | NB-100 (E1) | 225 | 0, 3, 5, 7, 9 | 2.94±0.47 | Pooled | 1.24±0.08 | 23.4±5.6 |
| | | | | | Independent | 0.810±0.001 | 1652±14 |
| | LAN-1 (E2) | 225 | 0, 3, 5, 7, 10, 12 | 1.93±0.21 | Pooled | 1.79±0.09 | 4.18±0.54 |
| | | | | | Independent | 1.22±0.09 | 88.1±31.2 |
| Carlsson & Nederman (1983) | Human glioma | | $^{137}Cs\ \gamma$, 0.98 Gy min$^{-1}$ | | | | |
| | U-118-MG (C1) | 650 | [10, 12.5, 15, 20] | 5.86±0.38 | | [1.88±0.37] | — |
| | Hamster embryonic lung | | | | | | |
| | CHEL (C2) | 350 | [25, 30, 35, 40, 45] | — | | [3.96±0.59] | — |
| | Human thyroid ca. | | | | | | |
| | HTh-7 (C3) | 700 | [10, 12.5, 15, 20] | — | | [1.73±0.42] | — |
| Durand (1975) | Chinese hamster | | $^{60}Co\ \gamma$, 1.4 Gy min$^{-1}$ | | | | |
| | V79-171 (D1) | 350 | [25, 30, 35, 40] | — | | [3.84±0.53] | — |
| | (D2) | 620 | [35, 40, 45, 50] | — | | [4.25±0.28] | — |
| Pourreau-Schneider & Malaise (1981) | Human melanoma | | $^{137}Cs\ \gamma$, 0.83 Gy min$^{-1}$ | | | | |
| | Nall (P) | 130 | [6, 9, 10, 11.5, 12] | — | | [1.26±0.72] | — |

[a]Data for two spheroid sizes analyzed together, as in original publication.

The same model was used to analyse curves of survival of clonogenic cells that had been irradiated *in situ* in the spheroid, which was then disaggregated either immediately or at some time thereafter. The majority of examples analysed here were for spheroids of 250 μm or less in diameter, for which the clonogenic cells had monophasic survival curves. For the present comparative analysis, the curves for SRU and clonogenic cell survival in larger spheroids have also been treated as monophasic. This procedure will tend to underestimate the true final Do of a biphasic curve, such as was fitted by West *et al.* (1984) to the survival curve for clonogenic cells of 600 μm diameter spheroids of V79-379A cells.

### Results

To our knowledge, only two publications give values for both spheroid growth delay (GD) and survival of clonogenic cells after several graded radiation doses (West *et al.*, 1984; Rofstad *et al.*, 1986). However, other publications contain GD curves that may be analysed to determine the range of values for sensitivity of SRU to be expected from this method. These are shown in Table I, together with details of experimental procedure.

In the study of West *et al.* (1984), spheroids were disaggregated immediately after irradiation, while Rofstad *et al.* (1986) varied disaggregation time between immediately and 18 h after irradiation. We have adopted the 18 h values from the latter experiments as this had been used previously in an analysis of spheroid 'cure' data from Rofstad *et al.* (Moore *et al.*, 1987). However for all tumour lines analysed here from both studies, repair of potentially lethal damage by clonogenic cells was said not to occur, i.e. the time of disaggregation did not change the shape of survival curves for clonogenic cells. The relationship of Do for clonogenic cells from spheroids and Do derived from growth delay curves, is shown in Figure 1a for the common-slope fits. For the ten sets of data for 6 different tumour lines there was a significant correlation ($r=0.910$, $P=0.00013$). The assumption of a $G_2$ block had relatively little effect on the results ($r=0.943$, $P<0.00001$; Figure 1a). The correlation between the two was less good when the Do's for SRU had been obtained by independently fitting the slopes of the regrowth curves in an experimental group ($r=0.607$, $P=0.415$, excluding the implausible value of 25.1 Gy in group W6). In previous studies, we analysed the relationship of Do for spheroid clonogenic cells to the Do derived from dose-incidence curves of spheroid 'cure', over comparable ranges of high dose (Moore & Hendry, 1984; Moore *et al.*, 1987). These seven sets of data have been included in Figure 1a; the correlation for these data alone was $r=0.986$, $P<0.0001$. For the combined data for local control and growth delay (common-fit), $r=0.912$, $P<0.0001$, the slope was insignificantly different from 1 (1.14, 95% c.l. 0.86 and 1.43) and the origin insignificantly different from 0 ($-0.0396$, 95% c.l. $-0.7$ and 0.5).

The correlation of E for clonogenic cells and for SRU measured by growth delay, was poor ($r=0.682$, $P=0.015$). The derived values for SRU were very commonly higher than those for clonogenic cells (Figure 1b). Broadly, although not universally, survival curves derived from the back-extrapolates of independently-fitted curves had lower Do's and higher extrapolation numbers than those where a common fit was applied (Table I). This could occur because of relatively shallow slopes for curves of regrowth after low or moderate doses of radiation (tending to increase the SF derived from the back extrapolation and hence the size of the shoulder region of the derived survival curve for SRU), or relatively steep slopes for regrowth after high doses. For the first four authors in Table I, and taking *all* regrowth curves into consideration, the VDT of spheroids regrowing

**Figure 1** (a) Relationship of Do for SRU derived from curves of spheroid growth delay (squares) or 'cure' (circles). Enclosed letters and numbers are keyed to **Table I**. Error bars are 1 s.e. of the mean; where not shown, errors were smaller than the symbols. Lines are least-squares linear regression fits to: GD data, not including the effect of a $G_2$ block (————), GD data, including the effect of a $G_2$ block (– – – –), cure data (–·–·–·–), GD data (no block) plus cure data (· · · · · ·); (b) Relationship of extrapolation number E for SRU derived from GD curves (log scale), and E for clonogenic cells (linear). Dashed line is the curve for equal values of E between 1 and 5. Details as for (a).

after irradiation was 41% higher than that of untreated controls. If, arbitrarily, these mixed data were divided into dose ranges, the relative VDT's were: for 0.5–3.0 Gy, 1.20; 3.5–6 Gy, 1.55; 6.5–9.0 Gy, 1.40; and 9.5–20 Gy, 1.11. The limitations to the validity of this exercise are obvious, but the evidence at present favours increased VDT's at moderate doses, and VDT's comparable to those of controls at high doses (for which the GD's are longest).

Values of Do and E for SRU derived from all published GD data of which we are aware, and of Do for SRU derived from 'cure' data, have been plotted as a function of spheroid size (Figure 2a, b). Values for Do ranged from 0.5 Gy (225 μm diameter spheroids of human neuroblastoma) to 4.2 Gy (620 μm spheroids of V79-171 Chinese hamster cells). In the GD studies of West *et al.* (1984), a trend was evident for derived Do's of SRU to increase with increasing spheroid size, seen most clearly for the rapidly-growing spheroids of V79-379A cells (VDT = 1.7 days at 600 μm diameter). With four exceptions, derived values of E fell within the range of 2 to 6. The exceptions, found in spheroids of 250 μm or less, ranged between 17 and 85 (Figure 2b).

**Figure 2** (a) Relationship of Do for SRU derived from GD or 'cure' curves, to the mean diameter of the spheroids at the time of irradiation. Details as for **Figure 1(a)**.; (b) Relationship of the extrapolation number E of SRU derived from GD curves (log scale), to the mean diameter of the spheroids at the time of irradiation (linear). Enclosed letters and numbers are keyed to **Table I**.

### Discussion

Multicellular spheroids grown *in vitro* are a tumour model in which variation in the subjects to be irradiated and irradiation conditions can be closely controlled (e.g. Durand, 1975; 1980). They should therefore be better models than macroscopic tumours to test rigorously how close is the correlation between overall response and clonogenic cell survival. Previous analyses of the shapes of curves of spheroid 'cure' *versus* dose for six different cell lines, yielded Do's for SRU only 20% higher on average than those for clonogenic cells (Moore & Hendry, 1984; Moore *et al.*, 1987; and Figure 1a, this paper). However, such studies can deal only with doses that are high in the context of spheroid cell survival. In order to generate a 'full' survival curve for TRU (e.g. Denekamp & Harris, 1975) or SRU (e.g. Wheldon *et al.*, 1985), analysis is made of curves of regrowth in volume after radiation. We have shown for spheroids growing in an air- or oxygen-equilibrated environment, that there was a good relationship between the final Do's for curves of survival of clonogenic and those for SRU derived from GD, when a common exponential slope was fitted to all the regrowth curves in a given experimental group. Notably in the series of West *et al.* (1984), the method reflected well the increase of Do of V79 clonogenic cells from 2 to 4 Gy as the spheroids enlarged (Figure 1a). Smaller differences in Do were less well resolved, e.g. for the eight sets of results where clonogenic cell Do fell between 1 and 2 Gy, $r=0.456$, $P=0.128$.

The above method of fitting regrowth curves imposed assumptions (ii) and (iii) (see **Methods**) on the data. It has already been noted that even in spheroids, *systematic* variations appear to exist in rates of regrowth after irradiation, being slower after moderate than after high doses. One possible explanation is that observed regrowth after lower doses is the product of incomplete clearance of dead cells and limited growth of 'doomed' cells (e.g. Thames *et al.*, 1986), in addition to clonal regrowth over the size range permitted by the assay conditions. If this surmise is correct, this artefact may limit the utility of low-dose regrowth curves in some spheroid lines. Fitting a common slope to all curves or setting all curves to the control growth rate minimises this effect and Wheldon (1980) has noted that relatively small changes in slope estimates (imposed in this case) should contribute only linearly to the error in the estimate of log survival of SRU.

A second potential source of systematic error is the assumption that clonal regrowth is initiated immediately after irradiation. We calculated the effect of a dose-dependent mitotic delay and found relatively little influence on derived Do (Figure 1a). However, Durand (1975) observed an apparently dose-dependent delay of up to 3 days in the regrowth of clonogenic cells of V79-171 spheroids. This would tend to lower the derived surviving fraction of SRU at high doses. However, he also found that clonal regrowth rate may be faster after high doses, so that the time to recovery to pre-treatment size would be somewhat reduced and the back-extrapolate of the subsequently-measured regrowth curve raised, tending to *increase* the derived surviving fraction.

This analysis of published data for multicellular spheroids demonstrates that in the majority of cases, overall response to radiation (growth delay or cure) reflects quantitatively the radiosensitivity of clonogenic cells. The value of such information is two fold. Firstly there is the interest of the relationship between different biological endpoints. Secondly it suggests an alternative approach to assay by clonogenic cell survival, for estimating the radiosensitivity of primary human tumour material. Such determinations are often hampered by inadequate cell yields, low plating efficiencies and problems in the production of single cell suspensions.

Not all spheroid lines are suitable for the present type of analysis, notably those in which volume does not decrease after irradiation in GD experiments but instead increases at a progressively slower rate with dose (see, for example, West *et al.*, 1984; Evans *et al.*, 1986). However, with such lines, the 'cure' endpoint might still be employed to estimate the survival characteristics of the SRU. Thus in those tumours for which there are no direct means of determining the radiosensitivity of clonogenic cells, the overall response to radiation of their spheroids might be usefully employed.

The authors are supported by the Cancer Research Campaign (UK).

### References

CARLSSON, J. & NEDERMAN, J. (1983). A method to measure the radio- and chemosensitivity of human spheroids. *Adv. Exp. Med. Biol.*, **159**, 399.

DENEKAMP, J. & HARRIS, S.R. (1975). Tests of two electron-affinic radiosensitisers *in vivo* using regrowth of an experimental carcinoma. *Radiat. Res.*, **61**, 191.

DURAND, R.E. (1975). Cure, regression and cell survival: A comparison of common radiobiological endpoints using an *in vitro* tumour model. *Br. J. Radiol.*, **48**, 556.

DURAND, R.E. (1980). Variable radiobiological responses of spheroids. *Radiat. Res.*, **81**, 85.

EVANS, S.M., LABS, L.M. & YUHAS, J.M. (1986). Response of human neuroblastoma and melanoma multicellular tumour spheroids (MTS) to single dose irradiation. *Int. J. Radiat. Oncol. Biol. Phys.*, **12**, 969.

GILBERT, C.W. (1969). Computer programmes for fitting Puck and probit survival curves. *Int. J. Radiat. Biol.*, **16**, 323.

MOORE, J.V. & HENDRY, J.H. (1984). Relationship of clonogenic cells and 'tumour-rescuing cells', modelled in irradiated spheroids *in vitro*. *Br. J. Radiol.*, **57**, 935.

MOORE, J.V., HENDRY, J.H. & HUNTER, R.D. (1983). Dose-incidence curves for tumour control and normal tissue injury, in relation to the response of clonogenic cells. *Radiother. Oncol.*, **1**, 143.

MOORE, J.V., WEST, C.M.L. & HENDRY, J.H. (1987). Radiation response of multicellular spheroids initiated from five human melanoma xenograft lines. *Br. J. Radiol.*, **60**, 302.

MOULDER, J.E. & ROCKWELL, S. (1984). Hypoxic fractions of solid tumours: Experimental techniques, methods of analysis, and a survey of existing data. *Int. J. Radiat. Oncol. Biol. Phys.*, **10**, 695.

NIAS, A.H.W. & FOX, M. (1968). Minimum clone size for estimating normal reproductive capacity of cultured cells. *Br. J. Radiol.*, **41**, 468.

POURREAU-SCHNEIDER, N. & MALAISE, E.P. (1981). Relationship between surviving fractions using the colony method, the LD50, and the growth delay after irradiation of human melanoma cells grown as multicellular spheroids. *Radiat. Res.*, **85**, 321.

RHEINHOLD, H.S. & DE BREE, C. (1968). Tumour cure rate and cell survival of a transplantable rat rhabdomyosarcoma following X-irradiation. *Eur. J. Cancer*, **4**, 367.

ROFSTAD, E.K., WAHL, A. & BRUSTAD, T (1986). Radiation response of multicellular spheroids initiated from five human melanoma xenograft lines. Relationship to the radio-responsiveness *in vivo*. *Br. J. Radiol.*, **59**, 1023.

SUIT, H.D. (1966). Radiation Biology: A basis for radiotherapy. In *Textbook of Radiotherapy*, Fletcher, G.H., (ed) p. 65. Kimpton, London.

THAMES, H.D., BROCK, W.A., BOCK, S.P. & DIXON, D.O. (1986). Effect of dose per fraction on the division potential of lethally irradiated plateau-phase CHO cells exposed to isoeffective fractionation regimens. *Br. J. Cancer*, **53** (Suppl. VII), 376.

THOMLINSON, R.H. & CRADDOCK, E.A. (1967). The gross response of an experimental tumour to single doses of X-rays. *Br. J. Cancer*, **21**, 108.

WEST, C.M.L., SANDHU, R.R. & STRATFORD, I.J. (1984). The radiation response of V79 and human tumour multicellular spheroids – cell survival and growth delay studies. *Br. J. Cancer*, **50**, 143.

WHELDON, T.E. (1980). Can dose-survival parameters be deduced from *in situ* assays? *Br. J. Cancer*, **41** (Suppl. IV), 79.

WHELDON, T.E., LIVINGSTONE, A., WILSON, L., O'DONOGHUE, J. & GREGOR, A. (1985). The radiosensitivity of human neuroblastoma cells estimated for regrowth curves of multicellular tumour spheroids. *Br. J. Radiol.*, **58**, 661.